# Secondary Metabolism and Defense Responses Are Differently Regulated in Two Grapevine Cultivars during Ripening

**DOI:** 10.3390/ijms22063045

**Published:** 2021-03-17

**Authors:** Giorgio Gambino, Paolo Boccacci, Chiara Pagliarani, Irene Perrone, Danila Cuozzo, Franco Mannini, Ivana Gribaudo

**Affiliations:** 1 Institute for Sustainable Plant Protection, National Research Council (IPSP-CNR), Strada delle Cacce 73, 10135 Torino, Italy; paolo.boccacci@ipsp.cnr.it (P.B.); chiara.pagliarani@ipsp.cnr.it (C.P.); irene.perrone@ipsp.cnr.it (I.P.); danila.cuozzo@unito.it (D.C.); franco.mannini@ipsp.cnr.it (F.M.); ivana.gribaudo@ipsp.cnr.it (I.G.); 2 Department of Agricultural, Forest and Food Sciences (DISAFA), University of Torino, Largo Paolo Braccini 2, 10095 Torino, Italy

**Keywords:** high-throughput sequencing, anthocyanin profile, flavonoid 3′,5′-hydroxylases, stilbene synthases, defense metabolism, *Vitis vinifera*

## Abstract

*Vitis vinifera* ‘Nebbiolo’ is one of the most important wine grape cultivars used to produce prestigious high-quality wines known throughout the world, such as Barolo and Barbaresco. ‘Nebbiolo’ is a distinctive genotype characterized by medium/high vigor, long vegetative and ripening cycles, and limited berry skin color rich in 3′-hydroxylated anthocyanins. To investigate the molecular basis of these characteristics, ‘Nebbiolo’ berries collected at three different stages of ripening (berry pea size, véraison, and harvest) were compared with *V. vinifera* ‘Barbera’ berries, which are rich in 3′,5′-hydroxylated anthocyanins, using transcriptomic and analytical approaches. In two consecutive seasons, the two genotypes confirmed their characteristic anthocyanin profiles associated with a different modulation of their transcriptomes during ripening. Secondary metabolism and response to stress were the functional categories that most differentially changed between ‘Nebbiolo’ and ‘Barbera’. The profile rich in 3′-hydroxylated anthocyanins of ‘Nebbiolo’ was likely linked to a transcriptional downregulation of key genes of anthocyanin biosynthesis. In addition, at berry pea size, the defense metabolism was more active in ‘Nebbiolo’ than ‘Barbera’ in absence of biotic attacks. Accordingly, several pathogenesis-related proteins, WRKY transcription factors, and stilbene synthase genes were overexpressed in ‘Nebbiolo’, suggesting an interesting specific regulation of defense pathways in this genotype that deserves to be further explored.

## 1. Introduction

Grapevine (*Vitis vinifera* L.) is one of the most economically important fruit crops, accounting for 7.45 million ha in 2016 (O.I.V., https://www.oiv.int/en/statistiques/ accessed on 12 Febraury 2021) and worldwide renowned for winemaking and fresh grape consumption. Grapevines are cultivated in different areas with a strong ability to adapt to diverse climates [1], exploiting the genetic diversity of the thousands of *V. vinifera* genotypes existing and the phenotypic plasticity of the species [2,3,4,5,6]. In recent years, the adaptation to different environments and ongoing climatic changes made the grapevine an interesting model species for investigating the genetic and molecular bases that underlie this phenotypic plasticity. In the last decade, following the availability of the grapevine genome [7], and with the great advances in high-throughput sequencing techniques, an increasing number of works explored the genotype x environment interactions in grapevine by comparing the same cultivar or clone in different environments [8,9,10,11], several genotypes in a single growing area [12,13], or genotype effects associated with biotic and abiotic stresses [14,15,16,17]. Modulation of the transcriptome is undoubtedly one of the driving factors for the grapevine adaptation capacity, which, in turn, can be controlled by epigenetic variations [18]. A few works have deepened the evolution of plant phenotypic plasticity associated with epigenetic regulation and changes in DNA methylation patterns in grapevine [19]. However, early evidence suggested that epigenetic modifications may affect transcriptomic plasticity and the interactions among grapevine cultivars, environment, and stressor agents [10,20,21,22]. Most of these works analyzed the characteristics of the berries and the complex metabolic processes associated with ripening, which are subjected to many variations based on different genotypes and are determinant for the quality of the final product, wine.

*V. vinifera* ‘Nebbiolo’ is one of the most important and ancient grape vines in the world [23,24]. Its area of cultivation is predominantly limited to northwestern Italy (hilly and mountainous areas of Piedmont, Aosta Valley, and Lombardy), although today it is also cultivated in California, Mexico, Australia, and other countries. The outstanding-quality wines obtained from ‘Nebbiolo’ grapes, such as Barolo and Barbaresco, are known and appreciated all over the world. ‘Nebbiolo’ is characterized by a large intra-varietal variability with several polymorphic clones showing different phenotypic characteristics [25] associated with genomic [26] and transcriptomic changes [11]. Genes involved in sugar signaling, anthocyanin biosynthesis, and other secondary metabolic pathways are differentially modulated among clones and vineyard, suggesting the existence of complex genotype x environment interactions that contribute to shape the agronomic features of different ‘Nebbiolo’ clones [11]. ‘Nebbiolo’ has specific phenotypic characteristics: it shows a long vegetative cycle, a long period of sugar accumulation during ripening, and a low level of anthocyanins in the skin. The last of these features, which is associated with an anthocyanin profile rich in 3′-hydroxylated anthocyanins, in particular peonidin-3-glucoside [27,28], determines the typical ruby color of ‘Nebbiolo’-based wines. A low total anthocyanin concentration could be a problem in the winemaking process, as it sometimes results in wines with insufficient color. Although some factors (agronomical practices, seasonal features, harvest conditions) can influence anthocyanin accumulation in the grape skin, and oenological practices can be adopted to reduce the loss of disubstituted anthocyanins during winemaking by oxidation, polymerization, and insolubilization processes [28], the anthocyanin content and profile are basically genotype-dependent [29]. The molecular basis of these differences between genotypes is not yet fully understood, although it has been suggested that transcriptional regulation of some key genes for anthocyanin biosynthesis, such as UDP-glucose:favonoid 3-O-glucosyl transferase (*VvUFGT*), anthoMATE transporter3 (*VvAM3*), glutathione S-transferase 4 (*VvGST4*), and flavonoid 3′,5′-hydroxylases (*F3′5′Hs*) can determine the levels of anthocyanins produced in some wine grape cultivars [30,31].

The analysis of genotype-based transcriptional differences can help to understand which and how specific metabolic pathways are differentially regulated during ripening [13]. To provide further deepening into this subject, ‘Nebbiolo’ berries collected at different stages of ripening were compared with *V. vinifera* ‘Barbera’ berries using transcriptomic and analytical approaches. ‘Barbera’ is the major red grape variety cultivated in northwestern Italy that contributes to the production of Protected Denominations of Origin (PDO) wines. ‘Barbera’ is a genotype characterized by high levels of anthocyanins with a profile rich in acylated and 3′,5′-hydroxylated anthocyanins, in particular malvidin-3-glucoside [32]. These berry characteristics are different from ‘Nebbiolo’, and as the two cultivars share the same cultivation environment, they make ‘Barbera’ an excellent candidate to compare with ‘Nebbiolo’.

In this work, we analyzed the transcriptome of ‘Nebbiolo’ and ‘Barbera’ berries at three developmental phases (berry pea size, véraison, and harvest), and we integrated molecular information with analytical data to dissect the hub functional metabolic pathways characterizing the two genotypes during ripening. Hence, we demonstrated that secondary metabolism and stress responses underwent different transcriptional modulations between ‘Nebbiolo’ and ‘Barbera’, pointing to a tight regulation of basal defense pathways specific of ‘Nebbiolo’.

## 2. Results and Discussion

### 2.1. Genotype Is the Main Factor that Controls the Berry Anthocyanin Profiling

In two seasons (2013 and 2014), the anthocyanin profiles of ‘Nebbiolo’ CVT71 and ‘Barbera’ CVT115, cultivated in contiguous rows within the same vineyard, were compared at a defined berry ripening level corresponding to TSS (Total Soluble Solids) values around 24° BRIX (Table 1). In ‘Nebbiolo’ grapes, the analytical results confirmed the prevalence of peonidin-3-glucoside followed by malvidin-3-glucoside and, in general, a low ratio of 3′,5′-hydroxylated anthocyanins versus 3′-hydroxylated forms [27,32,33]. ‘Barbera’ grapes had a high content of anthocyanins and a ratio between 3′,5′-hydroxylated and 3′-hydroxylated anthocyanins clearly in favor of the 3′,5′-hydroxylated forms with a prevalence in malvidin-3-glucoside, followed by delphinidin-3-glucoside and petunidin-3-glucoside (Table 1). The two climatic seasons were very different: summer 2014 was characterized by mild temperatures and more precipitations than summer 2013 (Figure S1) favoring a greater accumulation of anthocyanins and flavonoids in 2014. However, the comparison of the two cultivars indicated that differences in anthocyanin profiles linked to the year or to the genotype x year interaction were limited (Table 1). In ‘Nebbiolo’, a slight increase in total anthocyanin levels and a decrease in 3′,5′-hydroxylated anthocyanins were observed in 2014 in respect to 2013. Conversely, the comparison between 2014 and 2013 in ‘Barbera’ showed opposite results, highlighting a decrease in total anthocyanins and an increase in 3′,5′-hydroxylated forms. Yields of anthocyanin extractability had a tendency to increase in the second year of the trial, showing a strong genotype effect (Table 1); the 3′-hydroxylated anthocyanins of ‘Nebbiolo’ showed a higher extractability in comparison to ‘Barbera’ anthocyanins. Furthermore, in both seasons, the percentage of acylated anthocyanins was stable and more than double in ‘Barbera’ compared to ‘Nebbiolo’ (Table 1). Intra-specific variability associated with anthocyanin profiles of different genotypes has been largely documented [32,34,35]. Modest variations in anthocyanin profiles can be associated with environment, vintage, water stress [36,37], and changes in temperature [33,38]. However, the grape anthocyanin composition profile is mainly affected by the specific genetic characteristics of each genotype rather than environmental variables [39]. Profiling of di- and trihydroxylated anthocyanins indeed represents a straightforward chemotaxonomical way to easily classify colored-grape cultivars [32,40]. Accordingly, the collected data confirmed that a cultivar such as ‘Nebbiolo’ is characterized by a low percentage of 3′,5′-hydroxylated anthocyanins independently of vintage and growing site.

### 2.2. Overview of the Whole Transcriptome Changes Occurring in ‘Nebbiolo’ and ‘Barbera’ Grapes over Ripening

**Figure 1:**
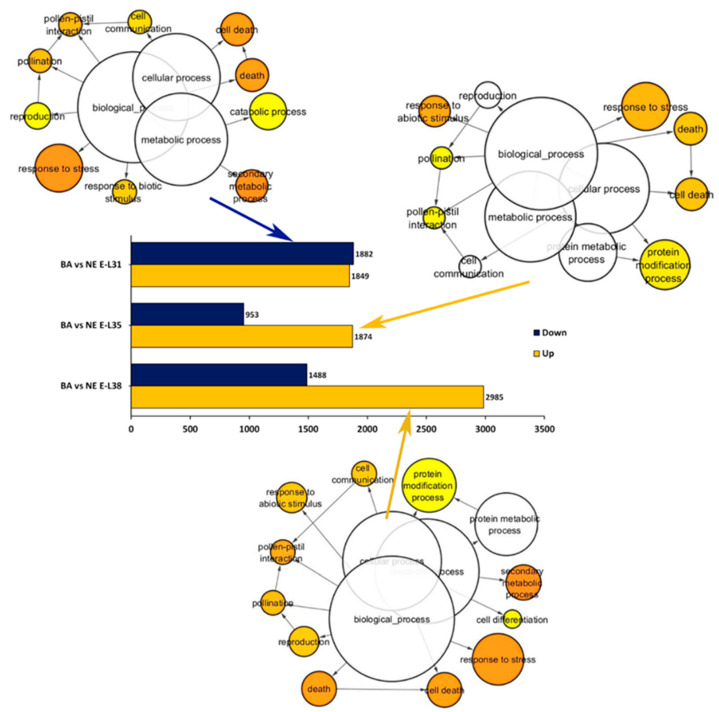
Transcriptome analysis of ‘Nebbiolo’ CVT71 (NE) and ‘Barbera’ CVT115 (BA) berries collected in 2013 at three developmental stages: pea size (E-L31), véraison (E-L35), and harvest at 24° BRIX (E-L38). The number of differentially expressed genes (DEGs), up (UP) or down (DOWN) regulated, is shown for each RNA-seq comparison near the bar charts. Significantly enriched Gene Ontology (GO) biological functional categories were identified for each group of DEGs belonging to downregulated genes at E-L31, upregulated genes at E-L35 and E-L38 in ‘Barbera’ using Cytoscape with the BINGO plug-in according to enrichment *p*-value (*p* ≤ 0.05).

High-throughput sequencing was performed on ‘Barbera’ and ‘Nebbiolo’ berries collected in 2013 at three developmental stages: pea size (E-L31), véraison (E-L35), and harvest (E-L38), following the classification of Coombe [41], respectively corresponding to stages 75, 81, and 89 of the extended BBCH scale by Lorenz et al. [42]. RNA-seq analysis produced an average of 23 million reads per sample that were aligned to the PN40024 reference genome with a mapping rate of 83.9% (Table S1). Differentially expressed genes (DEGs) were identified in three pairwise comparisons (‘Barbera’ vs. ‘Nebbiolo’ in E-L31, E-L35, and E-L38) using a *p*-value of 0.05% adjusted according to Benjamin–Hochberg. Of the 29,970 annotated genes, 14,458 were significantly differentially expressed in ‘Barbera’ and ‘Nebbiolo’ berries at one developmental stage at least. A fold-change (FC) cut-off was applied to analyze only the genes whose expression was |log2FC| ≥ 1, thus obtaining 7654 DEGs. At E-L31, 3731 DEGs were identified, 2827 at E-L35 and 4473 transcripts were differentially expressed at E-L38 in ‘Barbera’ and ‘Nebbiolo’ berries (Table S2). At E-L31, the number of up- and downregulated genes in the ‘Barbera’ vs. ‘Nebbiolo’ comparison was the same; however, Gene Ontology (GO) enrichment analysis conducted on transcripts belonging to downregulated genes in ‘Barbera’ (i.e., upregulated in ‘Nebbiolo’) indicated that response to stress, response to biotic stimulus, and secondary metabolism were the overrepresented functional categories (Figure 1).

Conversely, both at véraison and harvest, a higher number of upregulated genes was observed in ‘Barbera’, with an overrepresentation of transcripts involved in the response to stress, response to abiotic stimulus, protein modification, and secondary metabolism (Figure 1). Notably, many genes related to stress responses and secondary metabolism were upregulated in the ‘Nebbiolo’ green berries at E-L31, while during the subsequent developmental stages of ripening the expression of the same functional categories was strongly activated in ‘Barbera’. These data suggested that the genotype intrinsic features strongly control the modulation of the berry transcriptome during ripening, partially confirming previous observations obtained by comparing the transcriptome of several red and white grapevine varieties [13]. In particular, the transcriptional regulation of genes involved in secondary metabolism (e.g., phenylpropanoid/flavonoid biosynthesis) reflected the accumulation patterns of total anthocyanins in the berries over ripening. Nevertheless, other genes belonging to the phenylpropanoid pathway, such as the stilbene synthase genes (*STSs*), were overexpressed in ‘Nebbiolo’ at E-L31 in parallel with an interesting activation of several disease-resistance genes (Table S2).

### 2.3. Genes Associated with Secondary Metabolism Are Differently Reprogrammed in ‘Nebbiolo’ and ‘Barbera’ Grapes

**Figure 2:**
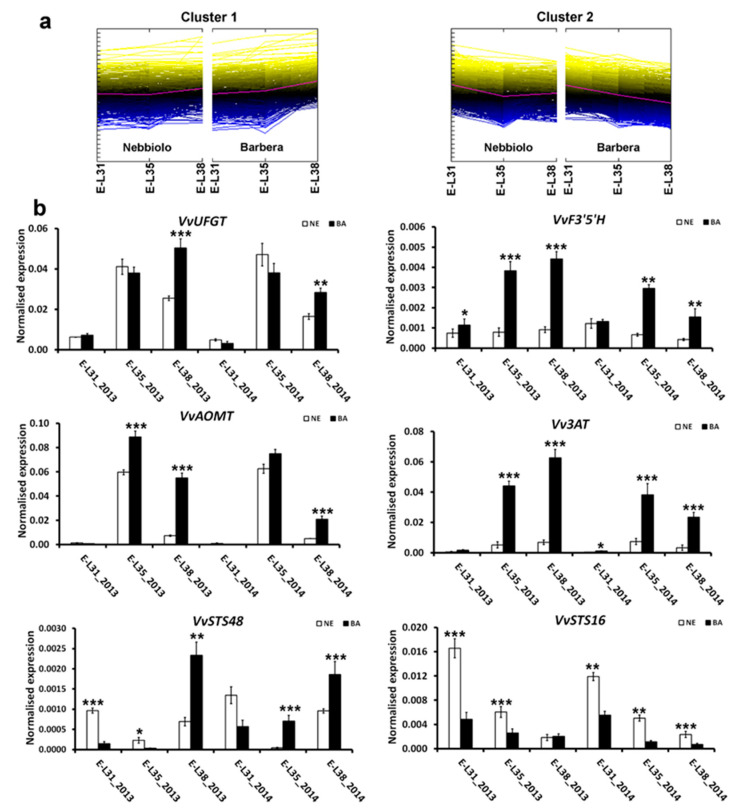
Transcriptional reprogramming of secondary metabolism genes in ‘Nebbiolo’ and ‘Barbera’ during ripening. ‘Nebbiolo’ CVT71 (NE) and ‘Barbera’ CVT115 (BA) berries were collected in 2013 and 2014 at three developmental stages: pea size (E-L31), véraison (E-L35), and harvest at 24° BRIX (E-L38). (**a**) Clusters of differentially expressed genes (DEGs) showing upregulated (Cluster 1) and downregulated (Cluster 2) genes in ‘Barbera’. (**b**) Results of candidate gene expression analysis performed by RT-qPCR assay. Cluster 1: anthocyanin O-methyltransferase (*VvAOMT*, VIT_01s0010g03510), UDP-glucose:flavonoid 3-O-glucosyltransferase (*VvUFGT*, VIT_16s0039g02230), flavonoid 3′,5′-hydroxylase (*VvF3′5′H*, VIT_06s0009g02810), anthocyanin acyltransferase (*Vv3AT*, VIT_03s0017g00870) and stilbene synthase *VvSTS48* (VIT_16s0100g01200). Cluster 2: stilbene synthase *VvSTS16* (VIT_16s0100g00840). *Ubiquitin* and *Actin* genes were used as endogenous controls for the normalization of transcript levels. Three independent biological replicates with three technical replicates each were used for analysis. Statistically significant differences between ‘Nebbiolo’ and ‘Barbera’ in each developmental stage were attested by analysis of variance for *p* ≤ 0.05 (*), *p* ≤ 0.01 (**), and *p* ≤ 0.001 (***).

Based on transcriptome results, secondary metabolism and stress responses were the functional categories the most modulated between the two cultivars; thus, we focused our survey on these groups of transcripts. The 439 DEGs identified in the three pairwise comparisons ‘Barbera’ vs. ‘Nebbiolo’ and involved in secondary metabolism were analyzed by hierarchical clustering (HCL), attesting that the DEGs were resolved to two major clusters (Figure 2). Cluster 1 was characterized by 295 genes upregulated in ‘Barbera’, in particular at harvest (Table S3). This cluster contained the most important genes involved in anthocyanin biosynthesis: chalcone synthase (*VvCHS*), chalcone isomerase (*VvCHI*), leucoanthocyanidin dioxygenase (*VvLDOX*), several flavonoid 3′,5′-hydroxylase (*VvF3′5′H*, VIT_06s0009g02830, VIT_06s0009g03000, VIT_06s0009g02840, VIT_06s0009g02810, VIT_06s0009g03010), several anthocyanidin 3-O-glucosyltransferase, UDP-glucose:flavonoid 3-O-glucosyltransferase (*VvUFGT*, VIT_16s0039g02230), anthocyanin acyltransferase (*Vv3AT*, VIT_03s0017g00870), and anthocyanin O-methyltransferase (*VvAOMT*, VIT_01s0010g03510). A selection of the most interesting genes belonging to Cluster 1 was analyzed by RT-qPCR, confirming the RNA-seq data on samples collected in 2013 (Figure S2). In addition, berries collected in 2014 were analyzed to verify the variation of the selected genes between the years. *VvUFGT*, directly involved in anthocyanin biosynthesis as well as the transcription factor *MybA1* [43,44], was overexpressed during ripening, as expected. In ‘Nebbiolo’, the *VvUFGT* expression peaked at E-L35 and then decreased, whereas in ‘Barbera’, the transcriptional levels of this gene remained high until harvest, suggesting that its continuous expression could positively influence the greater accumulation of anthocyanins in ‘Barbera’ than ‘Nebbiolo’ in both vintages (Figure 2b). The flavonoid 3′-hydroxylases (F3′Hs) compete with the flavonoid 3′,5′-hydroxylases (F3′5′Hs) for the same substrate to produce 3′-hydroxylated and 3′,5′-hydroxylated anthocyanins, respectively [45]. While *F3′Hs* are substantially not modulated during ripening, variations in the expression levels of *F3′5′Hs* affect the ratio between 3′,5′-hydroxylated and 3′-hydroxylated anthocyanins in different organs of the grape berry [30,31,46]. An example of the regulation of these genes is represented by *VvF3′5′H* (VIT_06s0009g02810), which was substantially not significantly modulated in ‘Nebbiolo’, showing an expression level stable at the different sampling times and in the two years, while in ‘Barbera’, it underwent a strong overexpression at E-L35 and E-L38 (Figure 2b) associated with the high accumulation of 3′,5′-hydroxylated anthocyanins (Table 1). *VvAOMT* is another important gene of the anthocyanin biosynthetic pathway, which is involved in the enrichment of methylated anthocyanin derivatives, such as malvidin and peonidin [47]. *VvAOMT* transcripts followed an expression pattern similar to that observed for *VvUFGT*, with a strong overexpression in ‘Barbera’ at the end of the season (Figure 2b). Furthermore, the expression trend of *Vv3AT*, which is involved in the synthesis of acylated anthocyanins [48], was well correlated with the high levels of acylated anthocyanins observed in ‘Barbera’, while in ‘Nebbiolo’, its expression underwent only a little increase during ripening (Table 1, Figure 2b). For all these genes, the expression levels in 2014 substantially mirrored the data obtained in 2013, confirming their higher expression in ‘Barbera’ than ‘Nebbiolo’. In 2014, a slight downregulation of the same genes was observed at harvest in both genotypes, which was likely associated with the milder temperatures and more abundant precipitation characterizing summer 2014 in comparison with 2013, in particular in September (Figure S1). However, in 2014, the expression differences between the two cultivars were similar to 2013, confirming that the transcriptional modulation of these genes is primarily affected by the genotype [31].

In Cluster 2, 144 genes for secondary metabolism were upregulated in ‘Nebbiolo’, in particular in green berries at E-L31 (Table S3). In this cluster, there were many genes involved in biotic and abiotic stress responses. For example, several S-adenosyl-L-methionine:salicylic acid carboxyl methyltransferase genes linked to the formation of methyl salicylate, which contributes to flavor and inter-plant communications during plant defense [49], were upregulated in ‘Nebbiolo’ (Table S3). However, these genes belong to a multigenic family whose transcriptional regulation is not uniform, and its function can only be determined after biochemical analyses [50]. In fact, some S-adenosyl-L-methionine:salicylic acid carboxyl methyltransferases were grouped in Cluster 1 and overexpressed in ‘Barbera’: this is the case of VIT_04s0023g02290, which is a marker of the early phases of ripening in grapevine [51].

The stilbene synthase genes (*STSs*) belong to another multigenic family strongly modulated in ‘Nebbiolo’ and ‘Barbera’ berries. All 31 *STS* genes, showing differential expression between the two cultivars, were upregulated in ‘Nebbiolo’ at E-L31. Along with ripening, a group of *STSs* was progressively downregulated in both genotypes, while a second group was upregulated at harvest with strong overexpression in ‘Barbera’ (Table S3). Examples of this differential regulation included *VvSTS16* (VIT_16s0100g00840) for the first group of *STSs* and *VvSTS48* (VIT_16s0100g01200) for the second group. For both genes, the same transcriptional regulation was confirmed in 2014 (Figure 2). Such differential modulation of *STS* transcripts likely implies different responses to metabolic signaling pathways and probably different *STS* biological roles. The *STS* gene family was previously characterized in grapevine [52]. *VvSTS16* and *VvSTS48* were classified in two different groups that are differentially expressed in response to diverse environmental stimuli. The accumulation of stilbenoids at harvest was previously reported [53], as well as possible crosstalk between the upregulation of *STSs* and the accumulation of anthocyanins in berries [13,54]. Interestingly, in the absence of biotic and abiotic stresses, grapes accumulate stilbenoids during ripening, with a variable efficiency based on the genotype. Accordingly, ‘Nebbiolo’ accumulated lower levels of resveratrol at harvest than other grapevine cultivars [53]. Our transcriptomic results confirmed these data; the *STS* genes were slightly modulated in ‘Nebbiolo’ at harvest compared to the expression peaks observed in ‘Barbera’ [55]. However, the strong activation of these genes in ‘Nebbiolo’ green berries at E-L31 did not affect the accumulation of stilbenoid compounds during subsequent ripening stages.

### 2.4. Regulation of Defense Metabolism Is Tightly Controlled by the Genotype Intrinsic Features

**Figure 3:**
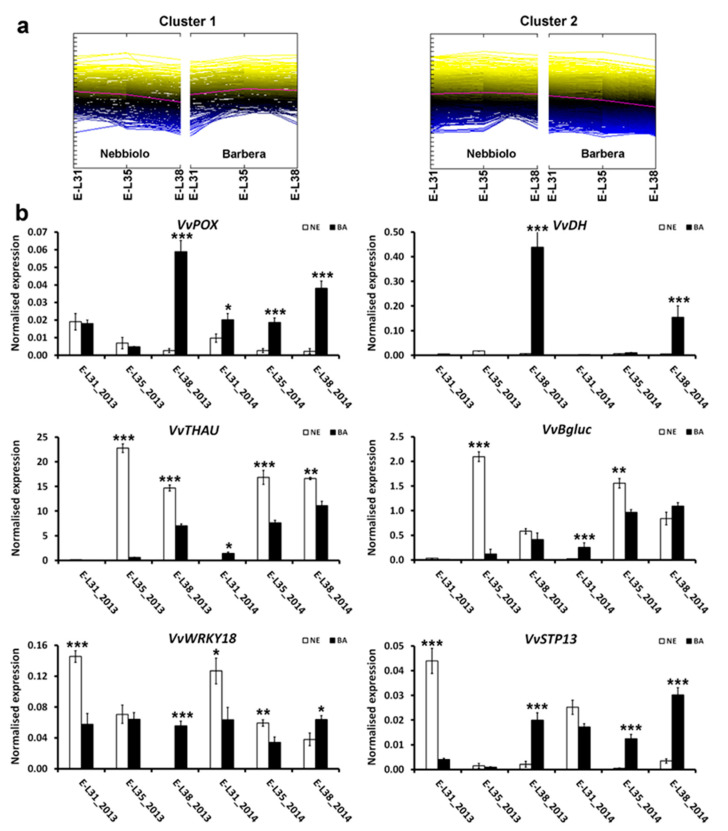
Transcriptional reprogramming of defense responses in ‘Nebbiolo’ and ‘Barbera’ during ripening. ‘Nebbiolo’ CVT71 (NE) and ‘Barbera’ CVT115 (BA) berries were collected in 2013 and 2014 at three developmental stages: pea size (E-L31), véraison (E-L35), and harvest at 24° BRIX (E-L38). (**a**) Clusters of differentially expressed genes (DEGs) showing upregulated (Cluster 1) and downregulated (Cluster 2) genes in ‘Barbera’. (**b**) Results of candidate gene expression analysis performed by RT-qPCR assay. Cluster 1: peroxidase (*VvPOX*, VIT_18s0072g00160) and Dehydrin (*VvDH*, VIT_04s0023g02480). Cluster 2: β1-3 glucanases (*Vvβgluc*, VIT_08s0007g06040) and thaumatin (*VvTHAU*, VIT_02s0025g04310). *VvWRKY18* (VIT_04s0008g05760) and sugar transporter *VvSTP13* (VIT_05s0020g03140). *Ubiquitin* and *Actin* genes were used as endogenous controls for the normalization of transcript levels. Three independent biological replicates with three technical replicates each were used for analysis. Statistically significant differences between ‘Nebbiolo’ and ‘Barbera’ in each developmental stage were attested by analysis of variance for *p* ≤ 0.05 (*), *p* ≤ 0.01 (**), and *p* ≤ 0.001 (***).

The 716 DEGs involved in stress responses and identified in the three pairwise comparisons ‘Barbera’ vs. ‘Nebbiolo’ were grouped into two major clusters by HCL. As for secondary metabolism, genes overexpressed in ‘Barbera’ at the end of the season (Cluster 1, 407 DEGs) clustered separately than genes overexpressed in ‘Nebbiolo’ at E-L31 (Cluster 2, 309 DEGs) (Table S3). At harvest, several stress-responsive transcripts, such as *STS* (see above), followed anthocyanin accumulation patterns. For example, genes involved in the detoxification of reactive oxygen species and abiotic stress as peroxidase *VvPOX* (VIT_18s0072g00160) and dehydrin *VvDH* (VIT_04s0023g02480) were strongly overexpressed in ‘Barbera’ at E-L38 in both years (Figure 3). *VvDH* is a gene activated in grapevine leaves and roots exclusively under water stress [56]. Its significant overexpression exclusive of ‘Barbera’ grapes could suggest a crosstalk between stress response and accumulation of secondary metabolites acting as antioxidant, such as anthocyanins. This possibility is further highlighted by the transcriptional increase of the abscisic acid biosynthetic gene 9-cis-epoxycarotenoid dioxygenase (VIT_02s0087g00930) in ‘Barbera’ during ripening (Table S2).

In ‘Nebbiolo’, several disease response genes were highly expressed during the early phases of ripening (E-L31 and E-L35), showing a modulation pattern similar to that reported for STSs (Table S3). Accordingly, several glutathione S-transferases, pathogenesis-related proteins, such as chitinases, thaumatins, and β1-3 glucanases, were upregulated in ‘Nebbiolo’ at E-L31 and/or E-L35, including *VvTHAU* (VIT_02s0025g04310) and *Vvβgluc* (VIT_08s0007g06040), and this transcriptional modulation is confirmed in 2014 (Figure 3). In addition, the overexpression of defense genes in ‘Nebbiolo’ was associated with the activation of specific transcription factors, such as *WRKY* genes, during the same ripening stages (Table S2). WRKY proteins were reported to control the basal defense responses of grapevine to abiotic and biotic stress in grapevine [57]. For instance, *VvWRKY18* was induced in ‘Gaglioppo’ scions when grafted onto rootstocks that stimulate defense responses [14]. In our dataset, *VvWRKY18* (VIT_04s0008g05760) was strongly expressed in ‘Nebbiolo’ at E-L31, and then its transcriptional rates decreased during ripening, while in ‘Barbera’, the expression levels of this gene were constant at all sampling times (Figure 3). Interestingly, the constitutive high basal expression of *VvWRKY18* in ‘Nebbiolo’ was also observed by analyzing the leaves of this cultivar in comparison to ‘Chardonnay’ [58].

In addition to specific transcription factors, sugars involved in the priming of plant defense responses can modulate defense-related genes [59]. For instance, the sugar transporter *VvSTP13* (VIT_05s0020g03140), which is involved in intracellular glucose uptake, was associated with defense responses in particular rootstock–scion combinations [14] and in response to fungal infection [58]. We found that, independently of the year, *VvSTP13* was overexpressed in ‘Nebbiolo’ berries at E-L31, while in ‘Barbera’, it was particularly induced at E-L38 (Figure 3). Based on the collected data, it thus emerges that, unlike ‘Barbera’, ‘Nebbiolo’ showed a constitutive basal activation of defense system-associated genes in green berries, which is independent of the presence of biotic agents. Accordingly, both ‘Barbera’ and ‘Nebbiolo’ vines had a similar virological profile (as they were only infected by the Grapevine rupestris stem pitting-associated virus, GRSPaV), were free of phytoplasmas, and berries were not attacked by fungal pathogens, as demonstrated in the previous work [11]. Therefore, during ripening, ‘Nebbiolo’ grapes showed a stronger constitutive expression of defense genes than ‘Barbera’, with the exception of the harvest stage, when other molecular regulatory signaling, mainly correlated with anthocyanin accumulation, probably occurred [13]. Notably, the high basal expression of stress-responsive genes in ‘Nebbiolo’ was also documented in leaves infected by powdery mildew and subjected to elicitor applications [16] as well as in leaves infected by viruses [58]. In addition, several cultivar-associated genes, specific of the ‘Nebbiolo’ genome and involved in response to pathogens, were previously identified [26]. This genotype-specific regulation of defense responses may also constitute an important ecological advantage, as it may result in reduced accumulation of harmful viruses, such as grapevine fanleaf virus [58], as well as in low titers and mild symptoms of Flavescence dorée phytoplasma [60,61], thus making ‘Nebbiolo’ more resilient than other cultivars (e.g., ‘Barbera’) to environmental and biotic constraints.

## 3. Material and Methods

### 3.1. Plant Material and Experimental Field

*V. vinifera* ‘Nebbiolo’ clone CVT71 and ‘Barbera’ clone CVT115 were cultivated in a vineyard located at Monforte d’Alba (northwestern Italy, 44°59′43.76″ N; 7°96′05.80″ E). Both cultivars were grafted onto Kober 5BB rootstock, and vines were vertically trained, Guyot pruned, and planted at a spacing of 0.9 m (within the row) × 2.4 m (between rows), resulting in an average density of about 4500 plants per ha. Experiments were conducted in two consecutive vegetative seasons (2013 and 2014). The vineyard was previously described [11] and used to study the clone by environment interplay in clones of ‘Nebbiolo’. In the present work, data of ‘Nebbiolo’ CVT71 clone grown in the V1 vineyard [11] were compared with those of ‘Barbera’ CVT115 clone cultivated in contiguous rows.

Only plants free from phytoplasma [62] and with a homogeneous virological status were used. Virus detection was carried out by ELISA and multiplex RT-PCR, as described by Gambino [63]: all vines resulted infected only by the GRSPaV, which is nearly ubiquitous and asymptomatic. Berry samples were collected at three stages during ripening: berry pea size E-L31, véraison E-L35, and harvest E-L38 (TSS around 24°BRIX) following the modified Eichhorn and Lorenz (E-L) system [41], corresponding to stages 75, 81, and 89 of the extended BBCH scale [42]. Berries were sampled from three randomized field plots, each constituted by seven vines per genotype, following the indications described in Pagliarani et al. [11]. Briefly, 200 berries for each biological replicate, stage, and genotype were collected from the upper, middle, and distal part of the bunch. Berries for metabolic analyses were used fresh, while for molecular analyses, they were frozen in liquid nitrogen and kept at −80 °C for one month until the time of RNA extraction.

### 3.2. Determination of Anthocyanin Profiles

Anthocyanin profiles were determined on three biological replicates of 30 berries randomly taken from the pool of 200 berries per genotype collected at E-L38. The skins were manually separated from the pulp and seeds, immersed in an ethanol buffer (pH 3.2), and incubated at 30 °C for 72 h to allow the extraction of phenolic compounds, as reported by Ferrandino and Guidoni [40]. The berry skin extracts were concentrated onto Sep-Pak C18 cartridges (Waters Corporation, Milford, MA, USA), eluted with methanol, and analyzed by liquid chromatography to determine anthocyanin profiles, according to Ferrandino et al. [64].

Contents of total flavonoids and total anthocyanins were determined on the grape skin extracts by spectrophotometry as described in Torchio et al. [65]. The anthocyanin extractability yield (%) was estimated after the extraction of homogenized skins in the above indicated ethanol buffer (pH 3.2) and centrifugation for 5 min at 3000× *g* at 20 °C. The content of anthocyanins on the obtained supernatants was used to evaluate the rate of skin anthocyanin extractability during maceration, as previously described in Rolle et al. [66].

TSS (°Brix) were measured on the musts at commercial harvest according to the methods proposed by the International Organization of Vine and Wine (O.I.V.-https://oiv.int/en/technical-standards-and-documents/methods-of-analysis/compendium-of-international-methods-of-analysis-of-wines-and-musts-2-vol accessed on 12 Febraury 2021).

Two-way analysis of variance (ANOVA) was conducted to test for the effect of each factor (genotype and year), and their interaction (SAS statistical software, version 8.2, SAS Institute, Cary, NC, USA). The Tukey’s honestly significant difference (HSD) post-hoc test was used to separate means (*p* ≤ 0.05, 0.01, and 0.001).

### 3.3. RNA Sequencing and Elaboration of the Data

Library preparation, sequencing, and data analysis were carried out as previously described [11,16]. Briefly, total RNA was extracted from 100 mg of deseeded berries using the Spectrum^TM^ Plant Total RNA extraction kit (Merck KGaA, Darmstadt, Germany). Only samples showing an RIN (RNA Integrity Number) value higher than 8, as assessed on an RNA 6000 Nano Labchip using a Bioanalyzer 1000 (Agilent Technologies, Santa Clara, USA), were submitted to sequencing and quantitative expression analyses.

Eighteen cDNA libraries (9 of ‘Nebbiolo’ and 9 of ‘Barbera’: 3 biological replicates for each genotype and developmental stage) were prepared from berries collected in 2013 using the TruSeq RNA Sample Prep Kit v2 (Illumina, San Diego, CA, USA) following the manufacturer’s instructions. Libraries were sequenced with an Illumina HiSeq 1000 sequencer (Illumina Inc., San Diego, CA, USA) by an external service (Functional Genomics Lab, Department of Biotechnology, University of Verona). Reads were aligned against the grape reference genome (12X genome of PN40024, https://urgi.versailles.inra.fr/Species/Vitis/Genome-Browser accessed on 12 Febraury 2021; [7]) using TopHat v.2.0.14 after removing low-quality reads (>50 bases with quality <7 or >10% undetermined bases). Cufflinks v.2.2.0 was used to normalize the expression of each transcript, which was calculated for each replicate as FPKM (fragment per kilobase of mapped reads). Differentially expressed genes (DEGs) were identified using the DESeq2 package (v.1.14.1, Buffalo, NY, USA) with a *p* value ≤ 0.01. Transcripts were annotated using the annotation V1 of the 12X draft grapevine genome [67] and grouped into functional gene classes according to the VitisNet GO annotations [68]. For hierarchical clustering (HCL) analysis, the MeV software (v4.9, Dana-Farber Cancer Institute, Boston, MA, USA) was used by applying the Pearson’s correlation distance of log2 transformed FPKM values derived from RNAseq data. The BiNGO 3.0 plug-in tool in Cytoscape (v3.2, U.S. National Institute of General Medical Sciences (NIGMS), Bethesda, MD, USA) was used for GO enrichment analysis [69]. Over-represented Plant GO slim categories were identified using a hypergeometric test with a significance threshold of 0.05.

### 3.4. Quantitative Real-Time PCR (RT-qPCR) Analysis

RNA samples collected in 2013 and 2014 from ‘Nebbiolo’ and ‘Barbera’ berries were analyzed by quantitative real-time PCR (RT-qPCR) to determine the transcript levels of a selection of genes (Table S4), following a previously reported protocol [14]. Briefly, RNA samples treated with DNase (DNase I, Invitrogen; Thermo Fisher Scientific, Waltham, MA, USA) were reverse-transcribed using the High-Capacity cDNA Reverse Transcription Kit (Applied Biosystems; Thermo Fisher Scientific). Real-time PCR assays were performed in a CFX Connect Real-Time PCR system (Bio-Rad Laboratories, Hercules, CA, USA), using SYBR Green (iQTM SYBR Green Supermix; Bio-Rad Laboratories,) with the following thermal cycling conditions: initial denaturation phase at 95 °C for 2 min, followed by 40 cycles at 95 °C for 15 s and 60 °C for 30 s. Ubiquitin (*VvUBI*) and Actin1 (*VvACT1*) were used as internal controls and the specific primers are reported in Table S4. Three independent biological replicates and three technical replicates were run for each RT-qPCR. Then, gene expression data were subjected to two-way ANOVA as described above. Statistically significant differences were highlighted at *p* ≤ 0.05, 0.01, and 0.001.

## 4. Conclusions

The comparison between the transcriptomes of ‘Nebbiolo’ and ‘Barbera’ at three crucial stages of berry development highlighted some specific transcriptional modulations of ‘Nebbiolo’, which were reflected in the physiological and biochemical characteristics of this genotype. At the molecular level, the typical anthocyanin profile observed in ‘Nebbiolo’ grapes, which are rich in 3′-hydroxylated anthocyanins, found some correspondence in the transcriptional downregulation of anthocyanin biosynthesis key genes, such as *VvUFGT*, *VvAOMT*, *Vv3AT*, and several *VvF3′5′Hs*. In ‘Barbera’, the same genes were strongly transcribed at harvest and favored the production of 3′,5′-hydroxylated and acylated anthocyanins. At harvest, these data were associated with an increase in the expression of some *STSs* and abiotic stress-responsive genes in ‘Barbera’ berries, which typically show higher accumulation of total anthocyanins than ‘Nebbiolo’. Such results confirm a cross-link between anthocyanin accumulation and stress responses, as suggested for other genotypes [13,31]. The main novelty of the work is the demonstration that in ‘Nebbiolo’, the basal defense metabolism was more active at early ripening stages (E-L31) and at véraison (E-L35) than in ‘Barbera’, even in the absence of biotic attacks. Interestingly, the defense-associated genes that were downregulated in ‘Nebbiolo’ at harvest (E-L38) did not correspond to the stress-responsive genes activated in ‘Barbera’ at the same phenological phase, evidencing a complex transcriptional regulation affecting transcripts belonging to the same gene family and functional category. Globally, these findings suggest that during ripening, ‘Nebbiolo’ mounted a specific transcriptional reprogramming of the basal defense metabolism that is different from that observed in other genotypes, which is a feature that is certainly worthy to be explored further in the future.

## Figures and Tables

**Table 1:** Total Soluble Solids (TSS, °Brix) determined at commercial harvest, total anthocyanins and flavonoids (mg kg^−1^), anthocyanin extractability, and anthocyanin profiles (percent) in the skin of grapes collected at 24 °BRIX from ‘Nebbiolo’ CVT71 and ‘Barbera’ CVT115 in 2013 and 2014. Data are means ± standard error (*n* = 21). Significance of genotype, year, and effects of the genotype × year interaction were tested for *p* ≤ 0.05 (*), *p* ≤ 0.01 (**), and *p* ≤ 0.001 (***); NS, not significant.

	2013		2014		Significance		
	Nebbiolo	Barbera	Nebbiolo	Barbera	Genotype	Year	Genotype x Year
Total Soluble Solids (TSS, °Brix)	23.95 ± 0.18	27.28 ± 0.50	23.07 ± 0.46	23.18 ± 0.55	**	***	**
Total flavonoids (mg kg^−1^)	2692 ± 741	4206 ± 210	3756 ± 224	2585 ± 96	NS	*	***
Total anthocyanins (mg kg^−1^)	749 ± 26.96	1719 ± 125.83	904 ± 25.98	1108 ± 59.17	***	***	***
Anthocyanin extractability (%)	61.53 ± 5.22	49.38 ± 0.88	76 ± 1.15	62 ± 2.74	***	***	NS
Delphinidin-3-*O*-glucoside	4.72 ± 0.33	21.16 ± 2.14	3.80 ± 0.10	18.5 ± 1.98	***	NS	NS
Cyanidin-3-*O*-glucoside	15.61 ± 0.95	15.6 ± 1.48	14.73 ± 1.47	7.99 ± 1.14	*	**	*
Petunidin-3-*O*-glucoside	4.10 ± 0.26	15.72 ± 1.38	3.43 ± 0.09	15.51 ± 0.29	***	NS	NS
Peonidin-3-*O*-glucoside	51.96 ± 1.66	11.33 ± 1.07	57.60 ± 0.44	7.70 ± 0.75	***	NS	**
Malvidin-3-*O*-glucoside	18.28 ± 1.91	24.58 ± 1.5	15.50 ± 1.15	38.71 ± 0.99	***	**	***
Delphinidin-acetylglucoside	0.09 ± 0.01	1.7 ± 0.16	0.07 ± 0.03	1.38 ± 0.48	***	NS	NS
Cyanidin-acetylglucoside	0.27 ± 0.02	1.15 ± 0.09	0.23 ± 0.03	0.47 ± 0.15	***	***	***
Petunidin-acetylglucoside	0.06 ± 0.01	1.48 ± 0.10	0.03 ± 0.03	1.23 ± 0.50	***	NS	NS
Peonidin-acetylglucoside	1.14 ± 0.07	0.72 ± 0.06	0.90 ± 0.00	0.38 ± 0.13	***	**	NS
Malvidin-acetylglucoside	0.55 ± 0.05	2.3 ± 0.08	0.37 ± 0.03	2.98 ± 1.26	***	NS	NS
Peonidin-caffeoylglucoside	0.11 ± 0.01	0.89 ± 0.18	0.10 ± 0.00	0.98 ± 0.16	***	NS	NS
Malvidin-caffeoylglucoside	0.32 ± 0.01	0.03 ± 0.01	0.30 ± 0.00	0.03 ± 0.01	***	**	*
Dephinidin *p*-coumaroylglucoside	0.14 ± 0.01	0.08 ± 0.02	0.10 ± 0.00	0.13 ± 0.07	NS	NS	NS
Cyanidin *p*-coumaroylglucoside	0.40 ± 0.02	0.93 ± 0.18	0.40 ± 0.06	0.58 ± 0.07	***	*	*
Petunidin *p*-coumaroylglucoside	0.09 ± 0.01	0.59 ± 0.07	0.10 ± 0.00	0.68 ± 0.12	***	NS	NS
Peonidin *p*-coumaroylglucoside	1.62 ± 0.08	0.49 ± 0.08	1.90 ± 0.06	0.34 ± 0.11	***	NS	*
Malvidin *p*-coumaroylglucoside	0.54 ± 0.06	1.25 ± 0.20	0.53 ± 0.03	2.41 ± 0.33	***	**	***
Total free trihydroxylated anthocyanins	27.10 ± 2.43	61.46 ± 1.53	22.77 ± 1.16	72.72 ± 1.12	***	NS	**
Total free dihydroxylated anthocyanins	67.57 ± 2.43	26.93 ± 1.46	72.32 ± 1.09	15.69 ± 1.09	***	NS	**
Total acylated anthocyanins	5.33 ± 0.16	11.61 ± 0.45	4.90 ± 0.07	11.59 ± 1.94	***	NS	NS
Trihydroxylated/dihydroxylated anthocyanins	0.40 ± 0.05	2.30 ± 0.19	0.32 ± 0.02	4.67 ± 0.27	***	***	***

## Data Availability

Raw sequences from the RNAseq libraries were deposited at the NCBI Sequence Read Archive under the project numbers PRJNA477842 for ‘Nebbiolo’ CVT71 (vineyard V1), and PRJNA691456 for ‘Barbera’ CVT115.

## Supplementary Materials

The following supplementary materials are available online at https://www.mdpi.com/1422-0067/22/6/3045/s1, References [70,71] are cited in the Supplementary Materials.
