# Supplementary material for: Secondary Metabolism and Defense Responses Are Differently Regulated in Two Grapevine Cultivars during Ripening
Source: Int J Mol Sci. 2021 Mar 17;22(6):3045. doi: 10.3390/ijms22063045 (PMC8002507; doi:10.3390/ijms22063045)

Secondary metabolism and defense responses are differently regulated in two grapevine cultivars during ripening

Giorgio Gambino*, Paolo Boccacci, Chiara Pagliarani, Irene Perrone, Danila Cuozzo, Franco Mannini, Ivana Gribaudo.

Figure S1: Climatic data. Monthly average values of Temperature (T, °C), Vapour Pressure Deficit (VPD, Pa KPa-1) and total rainfall (mm) in 2013 and 2014 in the area of the trial (Monforte d'Alba, Cuneo Province, North-West Italy). Bars represent standard error of the mean.

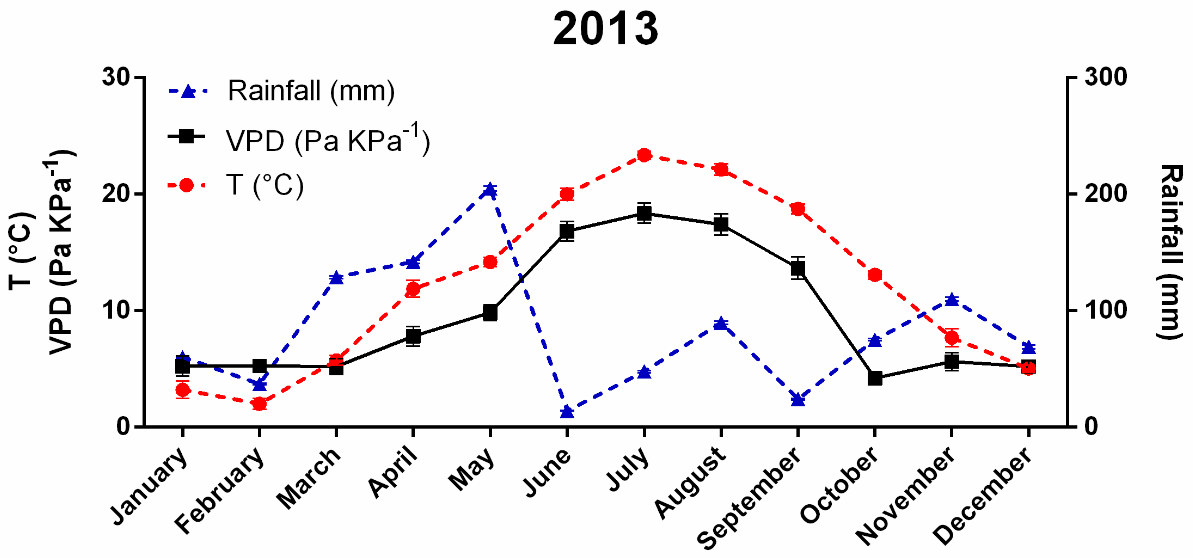

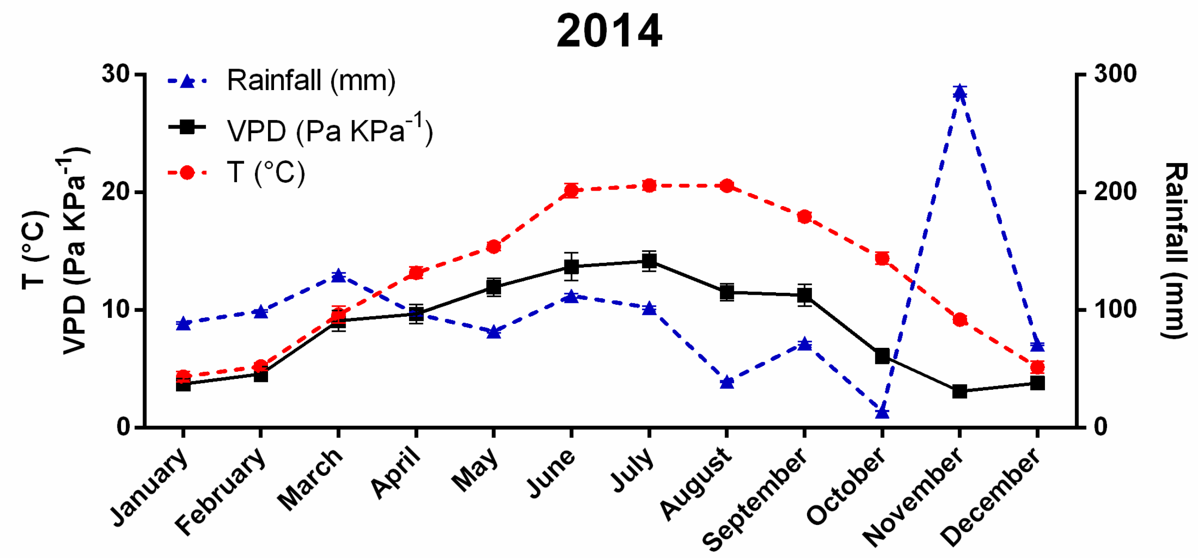

Figure S2: RNAseq vs RT-qPCR. Correlation between RNAseq (FPKM) and RT-qPCR results (normalized expression values) obtained from data of the expression analyses performed on transcripts (Table S4) used for validation of RNA-seq results. P value reported below the equation in the graph attests the significance of the regression analysis.

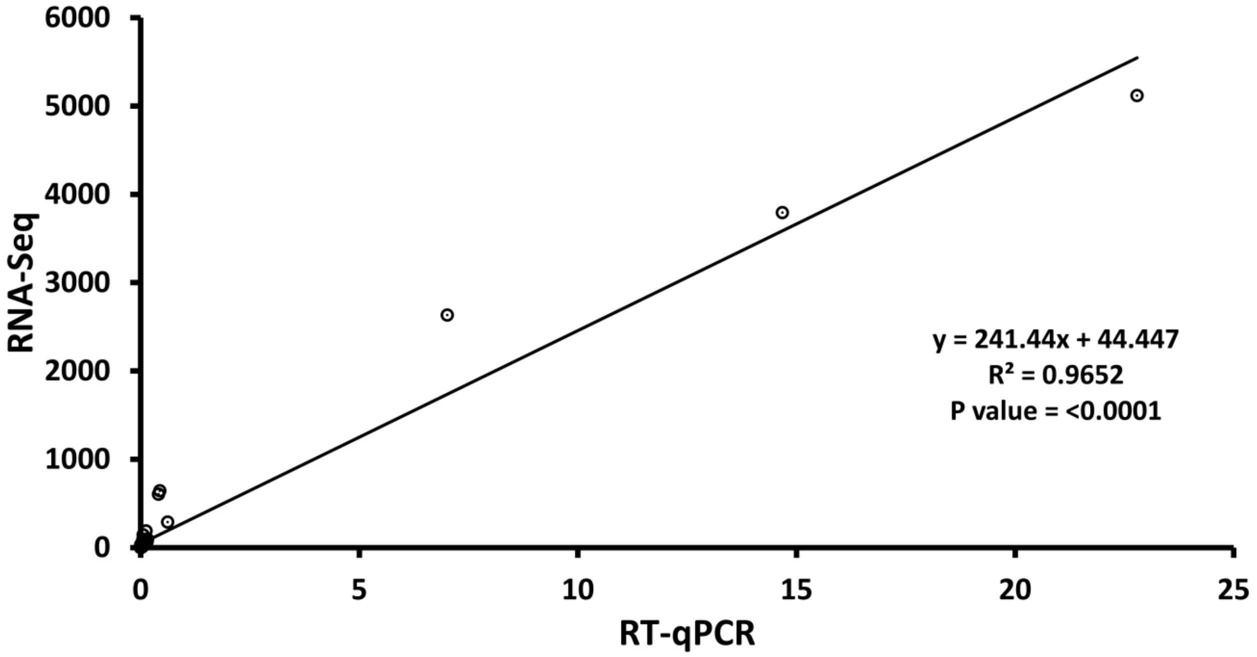

Supplement: Supplementary file 1 [file ijms-22-03045-s001.zip › supps resubmitted/Gambino Fig S1_S2.pdf]
